# Increased adenosine levels contribute to ischemic kidney fibrosis in the unilateral ureteral obstruction model

**DOI:** 10.3892/etm.2015.2177

**Published:** 2015-01-13

**Authors:** JIN TANG, XIANZHEN JIANG, YIHONG ZHOU, BING XIA, YINGBO DAI

**Affiliations:** Department of Urology, The Third Xiangya Hospital of Central South University, Changsha, Hunan 410013, P.R. China

**Keywords:** adenosine, α-smooth muscle actin, cytokines, renal interstitial fibrosis, unilateral ureteral obstruction model

## Abstract

Renal interstitial fibrosis (RIF) occurs as a result of chronic kidney disease (CKD) and is a common pathway leading to end-stage renal failure. Renal tissue hypoxia and ischemia are present during CKD. Adenosine (ADO) is an important signaling molecule induced under ischemic and hypoxic conditions. In the present study, the association between ADO and RIF was investigated using a mouse model, with the aim of obtaining important information relevant to the prevention and treatment of RIF. A unilateral ureteral obstruction (UUO) model of RIF was established in mice. A total of 44 male mice were randomly divided into sham, model and intervention groups, and samples were collected on days 1, 3, 7, and 14 after modeling. These were collected to detect hypoxia and changes in ADO concentration in obstructed renal tissue as well as to analyze the pathological changes and degree of RIF in the renal tissue. Changes in the levels of collagen deposition and profibrogenic factors in renal tissues were analyzed following intervention with an ADO receptor blocker. Following the UUO procedure, continuous hypoxia was present in the obstructed renal tissue, accompanied by an increased ADO concentration. Tubular injury and interstitial fibrosis progressively increased over time following the UUO procedure. The mRNA expression levels of tissue tumor growth factor β_1_ (TGF-β_1_) and α_1_(I) procollagen were significantly increased. Subsequent to the ADO pathway being blocked by 8-(*p*-sulfophenyl)-theophylline, tubular injury and interstitial fibrosis were reduced and the expression of related cytokines was decreased. Increased ADO levels were induced by hypoxia, causing the development of RIF. Following the blocking of the ADO pathway, renal damage was deferred and renal functions were protected.

## Introduction

The most prominent feature of chronic kidney disease (CKD) is renal interstitial fibrosis (RIF). The pathological changes associated with RIF include cellular changes, changes in the extra cellular matrix (ECM) and changes in growth factor interactions (1).

Studies have revealed that renal ischemia and hypoxia are caused by microvessel loss during CKD. Adenosine (ADO) is an important signaling molecule that is induced under ischemic and hypoxic conditions (2,3) and which plays a specific biological role following adenosine receptor (AR) binding on the cell surface. In renal tissues, ADO reduces renal blood flow by constricting afferent arterioles, thereby reducing renal transport load (4–8) and protecting short-term renal function. A previous study observed that adenosine deaminase (ADA)-deficient mice with prolonged exposure to high concentrations of ADO developed RIF lesions and renal damage, indicating that ADO plays an important role in promoting RIF damage (9).

Based on these facts, the present study considered the hypothesis that ADO may be an important signaling molecule in the development of RIF. It was hypothesized that under long-term ischemic and hypoxic conditions, intracellular proinflammatory cytokine release is induced by increased ADO concentrations in the renal tissues. Fibroblasts and collagen deposition are activated and regulated, which ultimately results in RIF and leads to kidney damage. Therefore, the present study selected the classic mouse unilateral ureteral obstruction (UUO) model mechanism of RIF. Following modeling, hypoxia of the renal tissue, ADO concentration changes and pathological changes in the obstructed renal tissues were observed at different time points in order to assess the degree of RIF. Changes in the deposition of related profibrogenic factors and interstitial collagen were detected through regulation of the ADO signaling pathway in order to investigate the association between ADO and RIF.

## Materials and methods

### Animals

The present study was carried out in strict accordance with the recommendations in the Guide for the Care and Use of Laboratory Animals of the National Institutes of Health, Second Edition (2011). The protocol was approved by the Committee on the Ethics of Animal Experiments of the Third Xiangya Hospital of Central South University (Changsha, China). All surgery was performed under sodium pentobarbital anesthesia and all efforts were made to minimize suffering. A total of 44 Kunming male mice with an average weight of ~40 g were randomly divided into three groups: Sham group (n=12), model group (UUO group; n=16) and intervention group (PT group; n=16). The mice in the intervention group were intraperitoneally injected with 10 mg/kg/day 8-(*p*-sulfophenyl)-theophylline (8-PT), a non-selective AR blocker, once each day following UUO surgery (1 time/day) (10). The mice in the model group were intraperitoneally injected with normal saline. On days 1, 3, 7, and 14 following each surgery, a number of mice were sacrificed, specifically, four each from the UUO and PT groups and three from the sham group. Prior to sacrifice, the mice were placed in metabolic cages for 24 h to enable the collection of urine. A solution of Hypoxyprobe^™^-1 (pimonidazole HCl; 60 mg/kg) from a Hyproxyprobe-1 kit (Hypoxyprobe, Burlington, MA, USA) was intravenously injected into the penile region of the mice in order to observe the degree of tissue hypoxia 1.5 h prior to the mice being sacrificed.

### UUO model

Mice were intravenously injected with 10% sodium pentobarbital (40 mg/kg). A 1.5-cm left upper quadrant midline incision was made to locate the left ureter. The renal pelvis near the ureter and the middle and upper junctions were ligated with no. 1 silk thread. The ureter between the two ligatures was cut. In the sham group, the left ureter was left free without ligation and the other steps were the same as for the UUO group.

### Quantitative polymerase chain reaction (qPCR)

The mouse cDNA sequence was obtained from GenBank. Primers were designed using Primer Premier software, version 3.0 (Premier Biosoft, Palo Alto, CA, USA) and were synthesized by ProMab (Richmond, CA, USA). Respective primer sequences are shown in Table I.

The total RNA in the renal tissue was extracted using TRIzol^®^ reagent (15596-026; Invitrogen Life Technologies, Carlsbad, CA, USA) and RiboLock^™^ ribonuclease inhibitor (EO0381; Thermo Fisher Scientific, Pittsburgh, PA, USA) was used to eliminate genomic DNA. The reverse transcription (RT) reaction was performed according to the instructions of the RevertAid^™^ H Minus First Strand cDNA Synthesis kit (K1631, Thermo Fisher Scientific). The mRNA transcript levels of tumor growth factor β_1_ (TGF-β_1_) and α_1_(I) procollagen were quantified using qPCR according to instructions provided with SYBR^®^ Green PCR Master Mix (4309155; Applied Biosystems, Carlsbad, CA, USA).

### Pathological analysis

The degree of tubular injury was scored by hematoxylin and eosin (H&E) staining; the degree of RIF was judged by Masson’s trichrome staining (11,12). Renal tissue TGF-β_1_ and α-smooth muscle actin (α-SMA) levels were detected at each time point by immunohistochemistry using anti-TGF-β_1_ (1:50, Bioss, Ltd., Woburn, MA, USA) and anti-α-SMA (1:200, Wuhan Boster Biological Technology, Ltd., Wuhan, China) antibodies. The degree of renal tissue hypoxia was semi-quantitatively determined using a Hypoxyprobe-1 kit (13).

A single-blind pathological examination was performed according to a multi-step procedure. Renal tissue samples were collected from the mice, paraffin-embedded and sectioned into 5-μm slices. From each renal sample four slices were randomly selected. Routine H&E and Masson’s trichrome staining was performed and the morphology observed using a microscope. Each slice was analyzed by the same individual with five non-overlapping fields randomly selected in each slice. Positive cells or areas were represented by an average optical density, separately calculated in the selected four parts of the renal tissue sample in each mouse. The average value of the four parts was calculated. The average optical density for the positive areas was automatically determined by Image-Pro Plus 6.0 software (Media Cybernetics, Rockville, MD, USA).

### High performance liquid chromatography (HPLC) assay

Approximately 1/3 of the left kidney was cut immediately upon being extracted from the mouse and preserved in a liquid nitrogen tank to determine the ADO concentration. The HPLC assay was performed on a reversed phase custom ocadecyl-silica (ODS) column (4.6×254 mm) with a detection wavelength of 260 nm at 30°C. Following adenine nucleotide extraction, the ADO concentration was designated the abscissa (x) and its corresponding peak area as the vertical axis (y). The regression equation was obtained; the ADO concentration was determined by conversion from the corresponding peak areas of the test sample.

### Statistical analyses

All data are expressed as the mean ± standard error of the mean and were analyzed for statistical significance using GraphPad Prism 5 software (GraphPad Software, San Diego, CA, USA). Student’s t-tests were applied for two-group analysis. The statistical significance of the differences in multiple groups of mice was assessed by analysis of variance (ANOVA). The comparison between two groups was performed using Tukey’s test. P<0.05 was considered to indicate a statistically significant difference.

## Results

### Continuous renal tissue hypoxia during RIF with increased ADO levels

**Figure 1:**
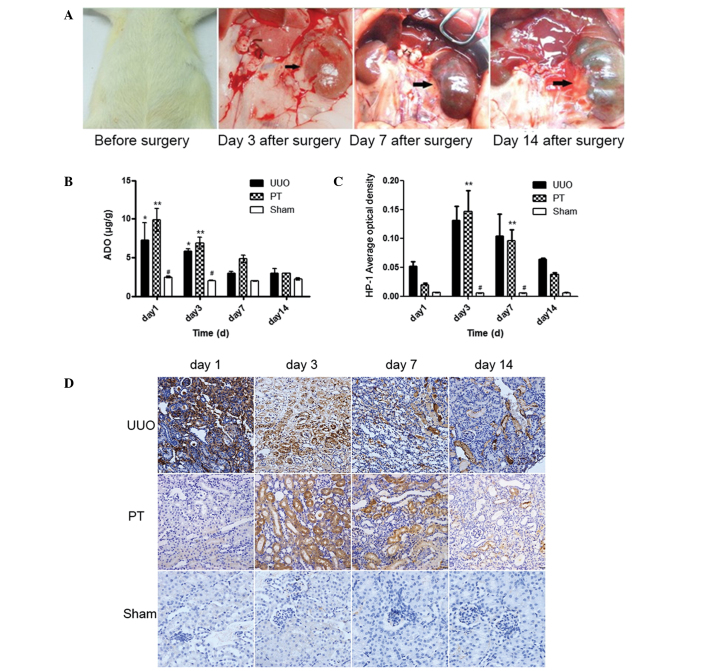
(A) Changes in the degree of hydronephrosis at different time periods. (B) The adenosine (ADO) concentration of each group. Data are expressed as the mean ± standard error of the mean (n=3 or 4). ^**^P<0.01 vs. the sham group; ^#^P<0.05 vs. the unilateral ureteral obstruction (UUO) group. (C) Changes in the degree of renal tissue hypoxia in each group. Data are expressed as the mean ± standard error of the mean (n=3 or 4). ^**^P<0.05 vs. the sham group; ^#^P<0.01 vs. the UUO group. (D) Immunohistochemical images of the obstructed renal tissue in each group determined by Hypoxyprobe-1 staining (magnification, ×200) injection. PT group, mice with UUO treated with 8-(*p*-sulfophenyl)theophylline.

Morphological changes in the kidney were observed on days 1, 3, 7 and 14 following modeling. As shown in Fig. 1A, hydronephrosis progressively increased over time following the UUO procedure.

### Hydronephrosis progressively increases as the duration of UUO is extended

The ADO concentrations in the renal tissue were determined through HPLC assays at different times following the UUO procedure. In the UUO and PT groups, the ADO concentrations on days 1 and 3 after modeling were revealed to be significantly higher than those in the sham group (P<0.05). In the PT group, the ADO concentrations on days 1, 3 and 7 after modeling were higher than those in the UUO group at the same time points. As the time subsequent to the UUO procedure extended, the ADO concentrations of the two groups converged on day 14 after modeling (Fig. 1B). These results indicated that after the AR was blocked by PT, extracellular ADO binding to the AR was reduced and metabolism slowed down, resulting in a progressive increase in the extracellular ADO concentration. However, following the UUO procedure, extracellular ADO concentration was decreased due to increased apoptosis and ATP depletion.

The degree of renal tissue hypoxia was measured following the intravenous injection of Hypoxyprobe-1 solution into the mouse penile region. Changes in the degree of hypoxia were observed following the UUO procedure. The degree of hypoxia in the UUO and PT groups was significantly higher than that in the sham group (P<0.05), particularly on days 3 and 7. On day 3, the degree of hypoxia peaked and subsequently gradually declined. No significant difference in the degree of hypoxia was identified between the PT and UUO groups (Fig. 1C and D). The brown-stained parts of the renal tissue represented the degree of renal tissue hypoxia. On day 3, the degree of hypoxia in the UUO and PT groups peaked and gradually declined thereafter.

### Promotion of RIF progress by increased ADO concentration is reduced by blocking the ADO pathway

**Figure 2:**
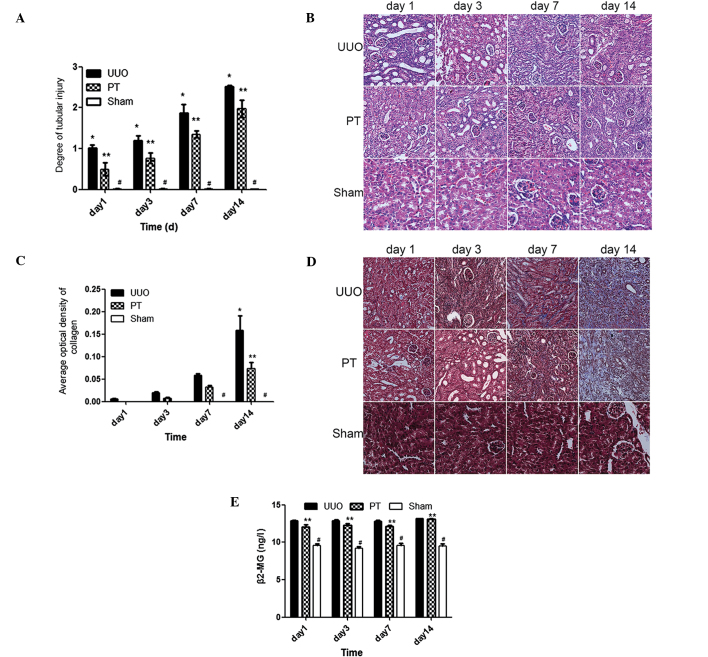
(A) Tubular injury score in each group. Data are expressed as the mean ± standard error of the mean (n=3 or 4). ^*^P<0.001 vs. the PT group; ^**^P<0.001 vs. the sham group; ^#^P<0.001 vs. the unilateral ureteral obstruction (UUO) group. (B) Hematoxylin and eosin staining images in obstructed renal tissue for each group (magnification, ×200). (C) Renal collagen content in each group. ^*^P<0.001 vs. the PT group; ^**^P<0.01 vs. the sham group; ^#^P<0.01 vs. the UUO group. (D) Masson’s staining images in obstructed renal tissue for each group (magnification, ×200). (E) β-2-microglobulin (β_2_-MG) concentration changes in 24-h urine for each mouse group; ^**^P<0.001 vs. the sham group; ^#^P<0.001 vs. the UUO group. (B and C) Data are expressed as the mean ± standard error of the mean (n=3 or 4). PT group, mice with UUO treated with 8-(*p*-sulfophenyl)theophylline.

H&E staining revealed that the tubular injury score in the UUO group was significantly higher than that in sham group at the same time point (P<0.001). Tubular dilation, accompanied by vacuolar degeneration, was observed on day 3 and progressively increased over time following the UUO procedure; this revealed a correlation between the post-UUO procedure time span and the degree of injury (P<0.0001). The PT group demonstrated a significantly reduced degree of tubular injury compared with that in the UUO group (P<0.05); however, the degree of injury remained higher than that in the sham group (Fig. 2A and B).

Tubular injury and the infiltration of interstitial inflammatory cells was observed in the UUO group on day 3. This progressively increased with time. In the PT group, the injury was significantly lower compared with that in the UUO group.

Collagen expression in the renal tissue was detected by Masson’s staining. On day 3, small amounts of collagen were observed in the renal interstitial tissue in the UUO group. These had significantly increased on day 7; on day 14, more collagen tracts were observed, revealing typical interstitial fibrotic changes. Collagen content in the UUO group on days 7 and 14 was significantly higher compared with that in the sham group (P<0.01). In the PT group, the collagen content was significantly lower than that in the UUO group (P<0.001) and significantly higher than that in the sham group (P<0.01; Fig. 2C and D).

The parts of the renal tissue stained blue represented collagen. Renal interstitial collagen content increased with time. At each time point, the collagen content of the PT group was lower than that of the UUO group.

The 24-h mouse urine sample was used for the detection of β-2-microglobulin (β_2_-MG). In the UUO group (Fig. 2E), the β_2_-MG concentration in the 24-h urine was significantly higher than that in the sham group (P<0.05) at each time point. In the PT group, the β_2_-MG concentration was lower than that in the UUO group; however, this difference was not statistically significant (Fig. 2).

### Role of the ADO signaling pathway

**Figure 3:**
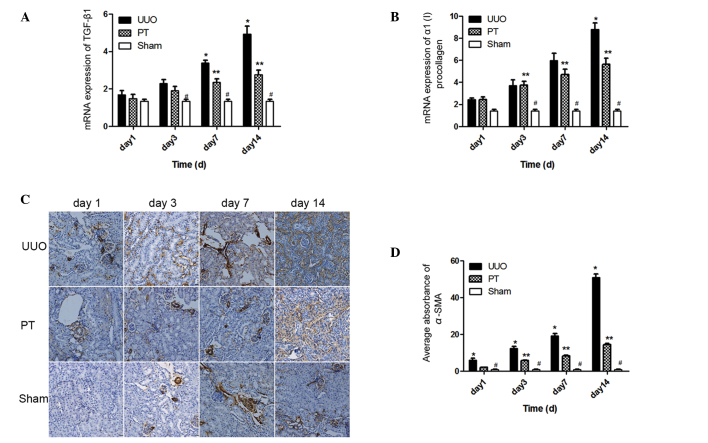
(A) Expression of TGF-β_1_ mRNA. The mRNA expression of tumor growth factor β_1_ (TGF-β_1_) in the unilateral ureteral obstruction (UUO) and PT groups significantly increased with time. Following blocking of the adenosine (ADO) signaling pathway, the mRNA expression of TGF-β_1_ in the PT group was notably lower than in the corresponding UUO group. ^*^P<0.01 vs. the PT group; ^**^P<0.01 vs. the sham group; ^#^P<0.01 vs. the UUO group. (B) Expression of α_1_(I) procollagen mRNA expression. The mRNA expression of α_1_(I) procollagen in the UUO and PT group was significantly increased with time. Following blocking of the ADO signaling pathway, mRNA expression of α_1_(I) procollagen in the PT group was notably lower than in the UUO group on days 7 and 14. ^*^P<0.001 vs. the PT group; ^**^P<0.001 vs. the sham group; ^#^P<0.001 vs. the UUO group. (C) Immunohistochemistry images of α-smooth muscle actin (α-SMA) in the obstructed renal tissue in each group (magnification, ×200). (D) Changes in α-SMA expression in the renal tissue of each group. The α-SMA expression in the UUO group significantly improved from the first to the 14th day following modeling. This notably decreased following the blocking of the ADO signaling pathway; ^*^P<0.01 vs. the PT group; ^**^P<0.01 vs. the sham group; ^#^P<0.001 vs. the UUO group. (A, B and D) Data are expressed as the mean ± standard error of the mean (n= 3 or 4). PT group, mice with UUO treated with 8-(*p*-sulfophenyl)theophylline.

TGF-β_1_ and α_1_(I) procollagen are two common profibrotic cytokines. The mRNA expression levels of these two cytokines significantly increased with time [TGF-β_1_, F=29.8; α_1_ (I) procollagen, F=32.85; P<0.0001] in the UUO and PT groups. On days 3, 4 and 7, the mRNA expression levels of TGF-β_1_ and α_1_(I) procollagen in the UUO group were significantly higher than those in the sham group (P<0.05). Following the blocking of the ADO signaling pathway, the mRNA expression levels of the two cytokines in the PT group were lower than those in the UUO group; however, the expression levels were higher than those in the sham group (P<0.05; Fig. 3A and B). Compared with the UUO group, the t-statistic of TGF-β_1_ mRNA expression on days 7 and 14 was 3.490 and 7.375, respectively (P<0.01); the t-statistic of α_1_(I) procollagen mRNA expression on day 14 was 5.705 (P<0.001). These results indicate that the ADO signaling pathway has a role in the regulation of these profibrotic cytokines, and that TGF-β_1_ and α_1_(I) procollagen expression was inhibited by ADO pathway blockage.

The expression and distribution of α-SMA in the renal tissue was dynamically observed by immunohistochemistry. In the sham group, only a small amount of α-SMA was expressed in the renal interstitial tissue blood vessels. In the UUO group, α-SMA was widely present in the mesenchymal and renal tubular epithelial cells. However, the expression was stronger than that in the sham group (P<0.001). With the prolonging of the time following UUO, α-SMA expression in the UUO group progressively increased. Following ADO pathway blockage, the distribution of α-SMA was consistent with that in the UUO group, but with a lower intensity (P<0.001). However, it remained higher than in the sham group on days 3, 7, and 14 (P<0.01; Fig. 3C and D). Thus, the ADO pathway regulated α-SMA expression in the renal tissue.

The parts of the renal tissue that were stained brown represented α-SMA expression. From the first day following modeling, the α-SMA expression in the UUO group was significantly higher than that in the other two groups and increased with time. The α-SMA expression in the PT group was lower than that in the UUO group at all time periods, but remained higher than that in the sham group.

## Discussion

RIF is the most important factor in CKD progression (14–16). Recent studies have indicated that tubulointerstitial hypoxia may stimulate kidney disease and it is considered to be one of the most important internal mechanisms of RIF (17–19). ADO is an important signaling molecule induced under ischemic and hypoxic conditions (2,3). In the present study, marked tubular injury was observed following the UUO procedure; the amount of interstitial collagen progressively increased with time. Along with renal damage, the β_2_-MG concentration in the UUO group was significantly higher than in the sham group at each time period. The degree of renal tissue hypoxia in the UUO group was significantly higher than in the sham group, which peaked on day 3 following UUO and subsequently decreased with time. A reason for this may be that in the time following the UUO procedure, normal tissue cells were replaced by large amounts of collagen, resulting in progressively decreased tissue protein content and the reduction of its ability to bind pimonidazole. Thus, the immunohistochemical staining decreased and the degree of hypoxia exhibited an unusual reduction. Tissue hypoxia was present during RIF in this part of the study.

Renal ischemia and hypoxia are conditions that are able to induce ADO. It was confirmed by HPLC assay that following the UUO procedure, the ADO concentration in the renal tissue was significantly higher than in the sham group and that this was most evident on days 1 and 3 after modeling. The concentration of ADO decreased with time after UUO but remained higher than in the sham group. The decreased ADO concentration was associated with increased apoptosis, ATP depletion and increased renal interstitial collagen levels. 8-PT was used in the current study as a non-selective AR blocker to block the ADO signaling pathway. Following the blocking of the ADO pathway, the ADO concentration in the renal tissue of the PT group was higher than that in the UUO group at the same time point. This result was related to the reduced binding of ADO with AR following blockage and a slowed metabolism, leading to extracellular ADO accumulation. Although the ADO concentration increased in the PT group, the degree of tubular injury, generation of renal interstitial collagen and β_2_-MG concentration in the 24 h urine significantly decreased compared with those in the UUO group. The ADO signaling pathway is associated with RIF and following the ADO signaling pathway blockage, RIF was effectively reduced and renal function was protected.

The RIF process not only controls cell activation and ECM deposition, but also maintains a close association with cytokine interactions (1). Presently, TGF-β_1_ is the most widely studied cytokine. Increased TGF-β_1_ levels have been revealed to have a causal association with tissue fibrosis (20). TGF-β_1_ has been confirmed as the strongest accelerator of ECM accumulation currently known. It functions through the TGF-β_1_/Smad signal transduction pathway and is recognized as a target for RIF therapy (21,22). In the current study, TGF-β_1_ mRNA content was detected using qPCR, and was higher in the UUO group than in the sham group. Following the blocking of the ADO pathway, the TGF-β_1_ mRNA level was significantly reduced. It was significantly lower in the PT group than in the UUO group (P<0.01), particularly on days 7 and 14. The above results indicate that hypoxia caused persistently elevated ADO concentrations while inducing the synthesis and secretion of large amounts of TGF-β_1_. Following the blocking of the ADO signaling pathway, TGF-β_1_ synthesis and secretion were inhibited, thus protecting renal function.

Renal fibrosis is caused by the excessive synthesis and decreased degradation of ECM (23), which is an important type of collagen constituting the structural renal tissue framework. The α-polypeptide chain is the basic subunit of collagen and α_1_(I) procollagen is one of the major components involved in collagen deposition. Thus, in the present study, the degree of renal fibrosis in renal tissue was assessed by the α_1_(I) procollagen content. From day 3 after modeling, α_1_(I) procollagen content in the renal tissue in the UUO group was consistently higher than in the sham group (P<0.05), indicating that large amounts of collagen had accumulated outside of the cells; thus, the renal tissue gradually experienced fibrosis. Following the blocking of the ADO pathway, the mRNA content of α_1_(I) procollagen was lower in the PT group than in the UUO group from day 7 post-modeling. The content continued to decrease and was significantly lower than that in the UUO group on day 14 (P<0.001). This result indicates that the ADO signaling pathway maintains a balance between the synthesis and degradation of the ECM and that this has positive effects in protecting renal function and repairing renal damage.

Fibroblasts are cells that are inherently present in the renal interstitium. They are relatively sparse under normal physiological conditions but greatly increase in number in fibrotic states. Fibroblasts are important in the development of RIF as they are ECM-secreting cells. When stimulated by inflammation, toxins or an immune response, fibroblasts in the renal interstitial region are activated, causing proliferative and phenotypic changes. The most representative change is the activation and transformation of fibroblasts into myofibroblasts (MFBs), which express α-SMA. The amount of collagen produced by MFB is 4–5 times as much as that produced by fibroblasts and this significantly influences the RIF process. Thus, a key step in RIF is the transformation of fibroblasts into α-SMA-expressing myofibroblasts (MFB). A study by Iwano *et al* reported that in the UUO model, ~36% of the fibroblasts were derived from the epithelial-interstitium transdifferentiation processes (24). In the present study, the expression of α-SMA in the renal tissue at different times was observed by immunohistochemistry. Following the UUO procedure, α-SMA expression in the renal interstitial and tubular tissue in the UUO group was significantly increased compared with that in the sham group (P<0.001). Following the blocking of the ADO pathway, α-SMA expression in the UUO group was significantly reduced compared with that in the UUO group (P<0.01). These results indicate that the ADO signaling pathway is associated with the proliferation and activation of the epithelial-mesenchymal transition (EMT) and fibroblasts. Notably, these processes were inhibited following ADO blockage.

In summary, the current study confirmed that ADO regulates the pathological processes of RIF using the mice UUO model. This provides an important experimental basis for further investigations of RIF pathogenesis. Furthermore, the association between AR types and RIF in the ADO signaling pathway, as well as the biological changes of important fibrotic effector cells, merits further study in order to provide novel antifibrotic therapy proposals for patients with CKD.

## Figures and Tables

**Table I:** Primer sequences.

Genes	Upstream and downstream primers (5′-3′)	Amplified fragment length (bp)	Temperature (°C)
α_1_(I) procollagen-F	GTTCTCCTGGCAAAGACGGA	199	58
α_1_(I) procollagen-R	CGGCCACCATCTTGAGACTT		
TGF-β_1_-F	AGGGCTACCATGCCAACTTC	168	58
TGF-β_1_-R	CCACGTAGTAGACGATGGGC		
α-SMA-F	GGACTCTGGAGATGGTGTGAC	167	58
α-SMA-R	CAATCTCACGCTCGGCAGTA		
GAPDH (mouse)-F	AACTTTGGCATTGTGGAAGG	132	58/59
GAPDH (mouse)-R	GGATGCAGGGATGATGTTCT		

TGF-β_1_, tumor growth factor β_1_; α-SMA, α-smooth muscle actin; GADPH, glyceraldehyde 3-phosphate dehydrogenase; F, forward; R, reverse.
